# Prediabetes and Fracture Risk Among Midlife Women in the Study of Women’s Health Across the Nation

**DOI:** 10.1001/jamanetworkopen.2023.14835

**Published:** 2023-05-23

**Authors:** Albert Shieh, Gail A. Greendale, Jane A. Cauley, Carrie A. Karvonen-Gutierrez, Arun S. Karlamangla

**Affiliations:** 1 Department of Medicine, David Geffen School of Medicine at University of California, Los Angeles; 2 Department of Epidemiology, School of Public Health, University of Pittsburgh, Pittsburgh, Pennsylvania; 3 Department of Epidemiology, School of Public Health, University of Michigan, Ann Arbor

## Abstract

**Importance:**

Whether prediabetes is associated with fracture is uncertain.

**Objective:**

To evaluate whether prediabetes before the menopause transition (MT) is associated with incident fracture during and after the MT.

**Design, Setting, and Participants:**

This cohort study used data collected between January 6, 1996, and February 28, 2018, in the Study of Women’s Health Across the Nation cohort study, an ongoing, US-based, multicenter, longitudinal study of the MT in diverse ambulatory women. The study included 1690 midlife women in premenopause or early perimenopause at study inception (who have since transitioned to postmenopause) who did not have type 2 diabetes before the MT and who did not take bone-beneficial medications before the MT. Start of the MT was defined as the first visit in late perimenopause (or first postmenopausal visit if participants transitioned directly from premenopause or early perimenopause to postmenopause). Mean (SD) follow-up was 12 (6) years. Statistical analysis was conducted from January to May 2022.

**Exposure:**

Proportion of visits before the MT that women had prediabetes (fasting glucose, 100-125 mg/dL [to convert to millimoles per liter, multiply by 0.0555]), with values ranging from 0 (prediabetes at no visits) to 1 (prediabetes at all visits).

**Main Outcomes and Measures:**

Time to first fracture after the start of the MT, with censoring at first diagnosis of type 2 diabetes, initiation of bone-beneficial medication, or last follow-up. Cox proportional hazards regression was used to examine the association (before and after adjustment for bone mineral density) of prediabetes before the MT with fracture during the MT and after menopause.

**Results:**

This analysis included 1690 women (mean [SD] age, 49.7 [3.1] years; 437 Black women [25.9%], 197 Chinese women [11.7%], 215 Japanese women [12.7%], and 841 White women [49.8%]; mean [SD] body mass index [BMI] at the start of the MT, 27.6 [6.6]). A total of 225 women (13.3%) had prediabetes at 1 or more study visits before the MT, and 1465 women (86.7%) did not have prediabetes before the MT. Of the 225 women with prediabetes, 25 (11.1%) sustained a fracture, while 111 of the 1465 women without prediabetes (7.6%) sustained a fracture. After adjustment for age, BMI, and cigarette use at the start of the MT; fracture before the MT; use of bone-detrimental medications; race and ethnicity; and study site, prediabetes before the MT was associated with more subsequent fractures (hazard ratio for fracture with prediabetes at all vs no pre-MT visits, 2.20 [95% CI, 1.11-4.37]; *P* = .02). This association was essentially unchanged after controlling for BMD at the start of the MT.

**Conclusions and Relevance:**

This cohort study of midlife women suggests that prediabetes was associated with risk of fracture. Future research should determine whether treating prediabetes reduces fracture risk.

## Key Points

**Question:**

Is prediabetes associated with increased fracture risk among midlife women?

**Findings:**

In this cohort study of 1690 midlife women without diabetes from the Study of Women’s Health Across the Nation cohort, relative to not having prediabetes at any visit before the menopause transition (MT), having prediabetes at every pre-MT visit was associated with a statistically significant 120% greater hazard for fracture during the MT and after menopause. This association was independent of bone mineral density at the start of the MT.

**Meaning:**

This study suggests that, for midlife women, prediabetes may be a risk factor for future fractures.

## Teaser

This cohort study uses data from the Study of Women’s Health Across the Nation to evaluate whether prediabetes before the menopause transition is associated with incident fracture during and after the menopause transition.

## Introduction

“Diabetic bone disease” and fractures are increasingly recognized as end-organ complications of diabetes.^1,2,3,4,5^ At the present time, whether prediabetes is also a risk factor for fractures is uncertain. Such an association is plausible; recently published data show that prediabetes is associated with lower bone turnover and worse trabecular bone microarchitecture.^6,7,8^ Moreover, at levels observed in individuals without type 2 diabetes, greater insulin resistance is associated with a lower bone mineral density (BMD),^9^ a lower trabecular bone score,^8^ lower indices of hip strength,^10,11^ and faster bone loss,^8^ all risk factors for fractures.

Elucidating whether prediabetes increases fracture risk is a step in understanding its clinical relevance. Although prediabetes is a risk factor for developing diabetes, clinicians disagree on how aggressively to treat it.^12,13^ Not every person with prediabetes develops type 2 diabetes, and prediabetes itself has not been definitively associated with end-organ complications. With respect to bone health, whether prediabetes is associated with fractures in the absence of prior or future progression to type 2 diabetes is unknown.

The first objective of this study was to examine whether prediabetes among midlife women is associated with subsequent fracture in the absence of type 2 diabetes. We focused on midlife, when women undergo the menopause transition (MT) and fracture risk accelerates.^14,15^ Because prediabetes may affect BMD,^16^ our second objective was to assess whether the potential association of prediabetes with fracture was independent of BMD.

## Methods

This cohort study used data collected between January 6, 1996, and February 28, 2018, in the Study of Women’s Health Across the Nation (SWAN), a multicenter cohort study of 3302 diverse, community-dwelling women. At the SWAN baseline visit, participants were 42 to 52 years of age and in premenopause (no change from usual menstrual bleeding) or early perimenopause (less predictable menstrual bleeding at least once every 3 months). Potential participants were excluded if they did not have an intact uterus and 1 or more ovaries or were using hormonal therapy or hormonal contraception. Women were recruited at 7 clinical sites: Boston, Massachusetts; Chicago, Illinois; Detroit, Michigan; Pittsburgh, Pennsylvania; Los Angeles, California; Newark, New Jersey; and Oakland, California. The SWAN Bone Cohort included 2365 women from 5 sites (excluding Chicago and Newark). Since study inception in 1996, 1 baseline visit and 16 follow-up visits have occurred at a median of 1.1 years (IQR, 1.0-1.4 years) between consecutive visits. All study volunteers provided written informed consent, and each site obtained institutional review board approval (University of Michigan; Massachusetts General Hospital; Rush University Medical Center; University of California, Davis, and Kaiser Permanente; University of California, Los Angeles; Albert Einstein College of Medicine; and University of Pittsburgh). This study followed the Strengthening the Reporting of Observational Studies in Epidemiology (STROBE) reporting guideline for observational studies.

### Samples

To be included in this analysis, SWAN Bone Cohort participants needed 1 or more study visits before the MT, could not be taking a bone-beneficial medication before the MT, could not have type 2 diabetes before the MT, and needed at least 1 study visit after the start of the MT (to permit observation for fractures). Bone-beneficial medications included hormone therapy, calcitonin, calcitriol, bisphosphonates, denosumab, and parathyroid hormone. We defined the start of the MT as the first visit in late perimenopause (less predictable menstrual bleeding at least once every 3-12 months). For women who transitioned directly from premenopause or early perimenopause to postmenopause (“skipped” late perimenopause), we defined the start of the MT as the first postmenopausal visit.

Starting with 2365 SWAN Bone Cohort participants, we excluded those with no pre-MT study visits (n = 34), women who started a bone-beneficial medication before the MT (n = 193), participants with type 2 diabetes before or at the start of the MT (n = 141), or women without follow-up between the start of the MT and time of fracture or censoring (n = 307). Our resulting analysis sample included 1690 women. Mean (SD) length of follow-up was 12 (6) years. Type 2 diabetes was defined as a fasting blood glucose level of 126 mg/dL or more (to convert to millimoles per liter, multiply by 0.0555) or taking metformin, sulfonylurea, meglitinide, thiazolidinedione, dipeptidyl peptidase 4 inhibitors, glucagonlike peptide-1 receptor agonists, or insulin. Participants were censored at initiation of bone-beneficial medications, new diagnosis of type 2 diabetes, or last follow-up. We excluded women who started a bone-beneficial medication before the MT, and censored subsequent initiators to prevent potential confounding by indication.

### Outcome

The outcome was time to first fracture after the start of the MT. Fracture occurrence and location were ascertained using standardized questionnaires at all study visits. Pre-SWAN fractures were recorded at the SWAN baseline visit. SWAN initiated fracture adjudication at follow-up visit 7; for the first 6 follow-up visits, fracture date was imputed as the midpoint between the participant’s previous and index visits. Since adjudication began, 95% of reported fractures were confirmed. Fractures were atraumatic if they occurred after a fall from a height of less than 15.2 cm and if they did not occur during a motor vehicle accident, rapid movement, playing sports, or from impact with heavy or fast-moving projectiles. We excluded craniofacial and digital fractures but included atraumatic and traumatic fractures because both fracture types are risk factors for subsequent fractures.^17,18^

### Primary Exposure

The primary exposure was prediabetes before the MT. We modeled our prediabetes exposure as the proportion of visits from the SWAN baseline visit through the last visit before the MT at which prediabetes was present. Criteria for prediabetes were a fasting blood glucose level between 100 and 125 mg/dL and not taking a diabetes medication. The prediabetes exposure was a continuous variable with values ranging from 0 to 1, which allowed us to capture how consistently a participant had prediabetes. For example, women who never had prediabetes had an exposure value of 0, and those who had prediabetes at every visit before the MT had an exposure value of 1. Women with prediabetes at 1 or more, but not all, pre-MT visits had exposure values between 0 and 1. Consistency of prediabetes is relevant because individuals with prediabetes may not meet glycemic criteria for prediabetes at every time point over a period of several years, with up to one-third returning to normal glucose regulation altogether.^19,20,21^

Fasting blood glucose was measured at 2 different central laboratories in SWAN. Through follow-up visit 7, glucose was measured at Medical Research Laboratories (Lexington, Kentucky) using a hexokinase-coupled reaction assay (Roche; intra-assay coefficient of variability, 1.6%). Subsequent glucose measurements were performed at the University of Michigan, Ann Arbor, using the ADVIA Chemistry Glucose Hexokinase assay (intra-assay coefficient of variability, 0.7%-0.9%). A between-laboratory calibration equation was developed using 565 randomly selected values across the range of glucose results. This equation was applied to convert Medical Research Laboratories results to corresponding University of Michigan values.

### Covariates

Analyses controlled for variables that were potentially associated with fracture. These variables included age at the start of the MT, body mass index (BMI; calculated as weight in kilograms divided by height in meters squared) at the start of the MT, cigarette use (yes or no) at the start of the MT, fracture before the MT (yes or no), race and ethnicity (Black, Chinese, Japanese, or White) by self-report, study site, exposure to bone-detrimental medications before the MT, and exposure to bone-detrimental medications during fracture observation. Bone-detrimental medications were oral or injectable glucocorticoids, aromatase inhibitors, gonadotropin-releasing hormone agonists, or antiepileptic medications. Exposure to these medications before the MT or during fracture observation was estimated as the proportion of visits during the respective time period at which use was reported. We adjusted for exposure to bone-detrimental medications, instead of excluding users, because very few SWAN participants took these drugs consistently over prolonged intervals.^15^

To address the second study objective, we adjusted for lumbar spine (LS) or femoral neck (FN) BMD at the start of the MT. Lumbar spine and FN areal BMD were measured by dual x-ray absorptiometry using Hologic Inc instruments. SWAN’s protocols for cross-site calibration, cross-calibration after machine upgrades, and quality control have been described previously.^22^

### Statistical Analysis

Statistical analysis was conducted from January to May 2022. We assessed all continuous variables for normality. Using the Spearman rank correlation, we examined the correlations between the proportion of visits before the MT with prediabetes and the mean glucose level across pre-MT visits with prediabetes.

We conducted 2 sets of primary analyses. The first (model 1) examined whether prediabetes before the MT was associated with subsequent fracture. We used Cox proportional hazards regression with the proportion of visits before the MT with prediabetes as the primary exposure and the time to first fracture after the start of the MT as the outcome. Covariates were age, BMI, and cigarette use at the start of the MT; exposure to bone-detrimental medication before the MT or after the start of the MT; fracture before the MT; race and ethnicity; and study site. Our second set of analyses assessed whether the hypothesized association of prediabetes with fracture was independent of BMD by adding to model 1 controls for LS (model 2A) or FN (model 2B) BMD. In all models, we added quadratic terms for the continuous prediabetes exposure and BMI to test for nonlinearity in their association with fracture. Neither quadratic term made statistically significant associations and were dropped from the final models.

Because the primary exposure is a continuous variable with values ranging from 0 to 1, the hazard ratio (HR) for prediabetes generated by the Cox proportional hazards regression model is the HR for prediabetes at all pre-MT visits (exposure = 1) relative to prediabetes at no pre-MT visit (exposure = 0). Because the primary exposure is continuous and had a linear association with fracture hazard, the estimated HR (for exposure = 1) can be converted to HRs for different exposure values between 0 and 1, corresponding to an individual woman’s proportion of pre-MT visits with prediabetes.

We conducted 2 sets of sensitivity analyses. First, we reran the models but used a binary exposure: any prediabetes before the MT (yes or no). Second, we reran models 1, 2A, and 2B using the original continuous prediabetes exposure but included only major nonvertebral fractures (defined in some osteoporosis therapy trials as fractures of the pelvis, distal femur, proximal tibia, ribs, proximal humerus, forearm, and hip) as outcomes.^23,24^

All analyses were performed using Stata, version 17 (StataCorp LLC). We used a 2-tailed significance level of .05.

## Results

### Participant Characteristics

Table 1 shows the characteristics of the full SWAN Bone Cohort (N = 2365) and our analysis sample (n = 1690; mean [SD] age, 49.7 [3.1] years; 437 Black women [25.9%], 197 Chinese women [11.7%], 215 Japanese women [12.7%], and 841 White women [49.8%]; mean [SD] BMI at the start of the MT, 27.6 [6.6]; mean [SD] LS BMD, 1.059 [0.143] g/cm^2^; mean [SD] FN BMD, 0.828 [0.133] g/cm^2^); both were similar. Women had a median of 3 visits (IQR, 1-6 visits) before the MT. The mean (SD) proportion of pre-MT visits with prediabetes was 0.070 (0.208). Fifty-six women (3.3%) had a fracture before the MT. Mean (SD) follow-up from the start of the MT to fracture or censoring was 12 (6) years. One-hundred thirty-six (8.0%) women sustained a fracture during the MT or in postmenopause. Of the 225 women with prediabetes, 25 (11.1%) sustained a fracture, while 111 of the 1465 women without prediabetes (7.6%) sustained a fracture. Thirty-three women were censored for initiating a bone-beneficial medication, and 94 for incident diabetes.

**Table 1.** Characteristics of Analysis Sample

Characteristic	Value at start of the MT^a^			
	SWAN Bone Cohort (N = 2365)	Full analysis sample (n = 1690)	Women without prediabetes before the MT (n = 1465)	Women with prediabetes at ≥1 visits before the MT (n = 225)
**Time-varying characteristics**				
Age, mean (SD), y	49.9 (3.2)	49.7 (3.1)	49.6 (3.1)	50.4 (3.0)
BMI, mean (SD)	28.2 (6.9)	27.6 (6.6)	27.1 (6.3)	31.7 (7.0)
Cigarette use (yes or no), No. (%)	273 (11.5)	221 (13.1)	188 (12.8)	33 (14.7)
Bone mineral density, mean (SD), g/cm^2^				
Lumbar spine	1.065 (0.147)	1.059 (0.143)	1.054 (0.143)	1.093 (0.141)
Femoral neck	0.834 (0.135)	0.828 (0.133)	0.821 (0.132)	0.869 (0.130)
**Fixed characteristics**				
Race and ethnicity, No. (%)				
Black	665 (28.1)	437 (25.9)	368 (25.1)	69 (30.7)
Chinese	250 (10.6)	197 (11.7)	166 (11.3)	31 (13.8)
Japanese	273 (11.5)	215 (12.7)	182 (12.4)	33 (14.7)
White	1177 (49.8)	841 (49.8)	749 (51.1)	92 (40.9)
Prediabetes status^b^				
Proportion of pre-MT visits at which prediabetes criteria were met, mean (SD)^c^	0.076 (0.208)	0.070 (0.208)	NA	0.524 (0.293)
Women with prediabetes at the following proportions of pre-MT visits, No. (%)				
>0 and <0.25	59/316 (18.7)	NA	NA	35 (15.6)
≥0.25 and <0.5	100/316 (31.6)	NA	NA	66 (29.3)
≥0.5 and <0.75	95/316 (30.1)	NA	NA	71 (31.6)
≥0.75 and <1	13/316 (4.1)	NA	NA	6 (2.7)
1	49/316 (15.5)	NA	NA	47 (20.9)
Fracture before the MT, No. (%)	87 (3.7)	56 (3.3)	47 (3.2)	9 (4.0)
Fracture after the start of the MT, No. (%)	189 (8.0)	136 (8.0)	111 (7.6)	25 (11.1)
Fractures after the start of the MT by fracture site, No. (%)				
Wrist	23 (12.2)	17 (12.5)	13 (11.7)	4 (16.0)
Hip	1 (0.5)	1 (0.7)	1 (0.9)	0
Spine	2 (1.1)	2 (1.5)	2 (1.8)	0
Pelvis	2 (1.1)	2 (1.5)	2 (1.8)	0
Ribs	9 (4.8)	6 (4.4)	3 (2.7)	3 (12.0)
Upper arm or shoulder	22 (11.6)	15 (11.0)	13 (11.7)	2 (8.0)
Leg above ankle	29 (15.3)	20 (14.7)	16 (14.4)	4 (16.0)
Ankle	35 (18.5)	27 (19.9)	22 (19.8)	5 (20.0)
Hand or foot	63 (33.3)	44 (32.4)	37 (33.3)	7 (28.0)
Other	3 (1.6)	2 (1.5)	2 (1.8)	0
Exposure to bone-detrimental medications before the MT^d^				
Women reporting use of a bone-detrimental medication at ≥1 study visit before the start of the MT, No. (%)	227 (9.6)	168 (9.9)	141 (9.6)	27 (12.0)
Proportion of pre-MT visits at which bone-detrimental medication users reported use, mean (SD)^c^	0.446 (0.295)	0.457 (0.295)	0.467 (0.301)	0.403 (0.259)
Exposure to bone-detrimental medications after the start of the MT^d^				
Women reporting use of a bone-detrimental medication at ≥1 study visit after the start of the MT, No. (%)	601 (25.4)	494 (29.2)	424 (28.9)	70 (31.1)
Proportion of visits after the start of the MT at which bone-detrimental medication users reported use, mean (SD)^c^	0.224 (0.193)	0.214 (0.187)	0.218 (0.191)	0.192 (0.150)

Abbreviations: BMI, body mass index (calculated as weight in kilograms divided by height in meters squared); MT, menopause transition; NA, not applicable; SWAN, Study of Women’s Health Across the Nation.

^a^ Start of MT defined as first visit in late perimenopause (less predictable menstrual bleeding once every 3-12 months), or first visit in postmenopause if participant transitioned directly from premenopause (no change in menstrual bleeding patterns) or early perimenopause (less predictable menstrual bleeding once every 1-3 months) to postmenopause.

^b^ Prediabetes defined as a fasting blood glucose level between 100 and 125 mg/dL (to convert to millimoles per liter, multiply by 0.0555), without use of diabetes medication.

^c^ Proportion of visits at which prediabetes criteria were met or bone-detrimental medication use was reported. This was a continuous variable with values ranging from 0 to 1. For example, women without prediabetes had values of 0. Women with prediabetes at all visits before the MT had values of 1. Women with prediabetes at 1 or more, but not all, pre-MT visits had values between 0 and 1.

^d^ Bone-detrimental medications were oral or injectable glucocorticoids, aromatase inhibitors, gonadotropin-releasing hormone agonists, or antiepileptic medications. Participants were considered users if they reported any use (regardless of duration).

A total of 225 participants (13.3%) had prediabetes at 1 or more study visits before the MT (Table 1). A greater proportion of Black, Chinese, or Japanese women were represented in this group. Also, among these 225 participants, the mean (SD) proportion of pre-MT visits with prediabetes was 0.524 (0.293), and prediabetes criteria were met in the majority of (≥50%) pre-MT visits for 124 women (55.1%) or all pre-MT visits for 47 women (20.9%) women. Seventy-three participants (32.4%) did not meet prediabetes criteria at any visit after their first prediabetes visit. Spearman rank correlations between the proportion of visits before the MT with prediabetes and the mean fasting glucose level at pre-MT visits with prediabetes was 0.83. At the start of the MT, the mean (SD) BMI among women with prediabetes was 31.7 (7.0), and the mean (SD) BMD was 1.093 (0.141) g/cm^2^ at the LS and 0.869 (0.130) g/cm^2^ at the FN. Among participants with prediabetes, the mean (SD) follow-up from the start of the MT to the time of fracture or censoring was 10 (6) years. Twenty-five women with prediabetes (11.1%) sustained a fracture, 1 was censored for starting a bone-beneficial medication, and 62 were censored for incident type 2 diabetes.

### Association of Prediabetes Before the MT With Subsequent Fractures

Having prediabetes more consistently before the MT was associated with greater rates of fracture during the MT or in postmenopause. In Cox proportional hazards regression, adjusted for age, BMI, cigarette use, bone-detrimental medications, fracture before the MT, race and ethnicity, and study site (model 1), relative to prediabetes at no pre-MT visit, prediabetes at every visit before the MT was associated with a 120% greater hazard of subsequent fracture (HR, 2.20 [95% CI, 1.11-4.37]) (Table 2). Compared with no prediabetes, HRs for prediabetes at 25%, 50%, and 75% of visits before the MT were 1.22 (95% CI, 1.03-1.45), 1.48 (95% CI, 1.05-2.09), and 1.81 (95% CI, 1.08-3.02), respectively. The observed fracture hazard of 6.3 per 1000 person-years for women with no prediabetes before the MT translated to an absolute increase of approximately 3 fractures per 1000 person-years for women who had prediabetes at half of the pre-MT visits and 7 fractures per 1000 person-years for women who had prediabetes at all pre-MT visits.

**Table 2.** Association of Prediabetes Before the MT With Subsequent Fracture During the MT and in Postmenopause^a^^,^^b^

Model	HR (95% CI) for fracture for prediabetes at all visits vs prediabetes at no visits before the MT (n = 1690)^a^	*P* value
Model 1: no BMD adjustment	2.20 (1.11-4.37)	.02
Model 2A: adjusted for lumbar spine BMD	2.24 (1.12-4.46)	.02
Model 2B: adjusted for femoral neck BMD	2.26 (1.13-4.49)	.02

Abbreviations: BMD, bone mineral density; HR, hazard ratio; MT, menopause transition.

^a^ Hazard ratios estimated using Cox proportional hazards regression with prediabetes before the MT as the primary exposure and time to fracture after start of the MT as the outcome. Start of the MT was defined as first visit in late perimenopause (less predictable menses once every 3-12 months), or first visit in postmenopause if participant transitioned directly from premenopause (no change in menstrual bleeding patterns) or early perimenopause (less predictable menstrual bleeding once every 1-3 months) to late perimenopause. Prediabetes was modeled as proportion of visits from the Study of Women’s Health Across the Nation baseline visit until last visit before the MT start that participants met prediabetes criteria. This was a continuous exposure with values ranging from 0 (prediabetes at 0 pre-MT visits) to 1 (prediabetes at all pre-MT visits). Women who had prediabetes at 1 or more, but not all, pre-MT visits had exposure values between 0 and 1. Model 1 was adjusted for age at start of the MT, body mass index at start of the MT, cigarette use at start of the MT, fracture before the MT, use of bone-detrimental medications before the MT, use of bone-detrimental medications during fracture observation, race and ethnicity, and study site. Models 2A and 2B additionally controlled for lumbar spine or femoral neck BMD, respectively. Because the primary exposure was continuous and had a linear association with fracture hazard, the estimated HR (for exposure = 1) can be converted to HRs for different exposure values between 0 and 1, corresponding to an individual woman’s proportion of pre-MT visits with prediabetes. To calculate the HR for a given proportion of visits before the MT with prediabetes, raise the HR presented to the desired proportion. Thus, the unadjusted HRs for prediabetes at 25%, 50%, or 75% of visits before the MT were 1.21 (2.20^0.25^), 1.49 (2.20^0.5^), and 1.81 (2.2^0.75^), respectively.

^b^ Of 1690 participants, 136 sustained a fracture. Fracture sites included wrist (n = 17), hip (n = 1), spine (n = 2), pelvis (n = 2), ribs (n = 6), upper arm or shoulder (n = 15), leg above ankle (n = 20), ankle (n = 27), hand or foot (n = 44), and other (n = 2).

Adding additional controls for LS (model 2A) or FN (model 2B) BMD at the start of the MT did not substantially alter the magnitude or statistical significance of the association between prediabetes and fracture. Hazard ratios for prediabetes at all pre-MT visits vs no pre-MT visits in models 2A and 2B were 2.24 (95% CI, 1.12-4.46) and 2.26 (95% CI, 1.13-4.49), respectively (Table 2).

### Sensitivity Analyses

Our first set of sensitivity analyses used a binary prediabetes exposure (any prediabetes before the MT) instead of the proportion of visits before the MT with prediabetes. The HR for any prediabetes before the MT were 1.94 (95% CI, 1.23-3.06) unadjusted for BMD, 2.00 (95% CI, 1.27-3.15) adjusted for LS BMD, and 2.02 (95% CI, 1.28-3.19) adjusted for FN BMD.

In our second set of sensitivity analyses, we retained the continuous prediabetes exposure from our primary models but considered only major nonvertebral fractures (n = 63) as outcomes. The HR for prediabetes at all vs no pre-MT visits was 2.58 (95% CI, 0.99-6.83) adjusted for BMD, 2.65 (95% CI, 1.00-7.03) adjusted for LS BMD, and 2.71 (95% CI, 1.02-7.17) adjusted for FN BMD.

## Discussion

In this longitudinal cohort study of the association between prediabetes in midlife women and fractures, we found that prediabetes before the MT was associated with more subsequent fractures; having prediabetes at 50% or 100% of pre-MT visits was associated with 49% and 120% greater hazard, respectively, for fracture during the MT and in postmenopause. The observed fracture hazard of 6.3 per 1000 person-years for women with no prediabetes before the MT translated to an absolute increase of approximately 3 and 7 fractures per 1000 person-years for women who had prediabetes at half of the pre-MT visits and all pre-MT visits, respectively. This increase in fracture risk was specific to prediabetes and was not associated with overt type 2 diabetes. Similar to type 2 diabetes,^1,2,3,4,5^ however, the prediabetes-fracture association was independent of BMD, suggesting that pathways other than BMD are associated with this risk.

Prior longitudinal analyses of prediabetes and fracture are scarce, with conflicting results.^25,26,27^ Among 5032 men and women (mean age, 74 years) from the third National Health and Nutrition Examination Survey (NHANES III) and NHANES 1999-2004, prediabetes was associated with nonsignificant trends toward greater risk of noncraniofacial factures among Mexican American (HR, 1.20 [95% CI, 0.96-1.51]) and White (HR, 1.42 [95% CI, 0.72-2.82]) participants.^25^ Among 5994 men (mean age, 73 years) from the Osteoporotic Fractures in Men study, impaired fasting glucose was not associated with subsequent nonvertebral fractures, before (HR, 0.95 [95% CI, 0.94-1.34]) or after (HR, 1.04 [95% CI, 0.89-1.21]) adjustment for BMD.^26^ Last, among 5 761 785 older South Korean adults (mean age, 61 years), prediabetes was associated with a modestly greater risk of subsequent hip fracture (HR, 1.17 [95% CI, 1.11-1.23]).^27^

Our analysis differed from previously published studies in 3 major ways. First was our prediabetes exposure, which models the consistency of prediabetes over time. This contrasts with prior studies that assessed glycemic status at 1 time point. Our finding that the proportion of visits with prediabetes had a positive, linear association with fracture suggests that more consistent prediabetes could be detrimental to fracture risk. Greater prediabetes consistency may indicate more severe prediabetes (inferred by the positive correlation between prediabetes consistency and fasting blood glucose) and longer duration of prediabetes Second, whereas previous studies examined older adults, we analyzed midlife women undergoing the MT, a period when insulin resistance and fracture risk accelerate.^14,15^ It may be easier to discern a prediabetes-fracture association during the MT because physiological changes are larger. Third, we designed our analysis such that the observed association of prediabetes with fracture could not be attributed to future progression to type 2 diabetes; prior studies did not censor participants at first type 2 diabetes diagnosis.

Our results add to a body of data suggesting that prediabetes is detrimental to bone health. Several recent SWAN analyses showed that prediabetes was associated with worse trabecular bone microarchitecture^8^ and that greater insulin resistance was associated with diminished bone strength^10^ and faster BMD loss.^22^ In other cohorts, prediabetes was associated with lower bone turnover,^6,7^ which can contribute to an accumulation of advanced glycation end products in bone and suboptimal bone material properties.^28,29,30,31^

Establishing whether prediabetes is a risk factor for fractures has potential public health implications. Nearly 1 in 3 US adults has prediabetes,^32^ but clinicians debate the need to treat it, in part because prediabetes has not been directly linked to end-organ complications.^13^ Our results suggest that more consistently having a fasting blood glucose level in the prediabetes range before the MT may be an independent risk factor for fracture. In our cohort of midlife women, most fractures occurred at nonhip, nonvertebral sites; these fractures can confer up to a 2-fold greater risk of subsequent vertebral or hip fracture.^33,34,35,36^ Thus, for this population, prediabetes before the MT may be an early, modifiable risk factor for fracture.

### Limitations

This study has several limitations. First, to maximize our fracture outcomes, we included both minimal trauma and traumatic fracture in a composite outcome. We justify this approach given the increasing recognition that both fracture types are associated with future fractures.^17,18^ Some randomized clinical trials assessing the antifracture efficacy of osteoporosis treatments now include minimal trauma and traumatic fractures as outcomes.^37^ Second, the rates of prediabetes and fractures differed by race and ethnicity. However, we did not have sufficient power to test whether the association between prediabetes and fractures differed by race and ethnicity.

## Conclusions

In this cohort study of midlife women, prediabetes before the MT was associated with greater risk of subsequent fractures during the MT and after menopause, independent of BMD. Because midlife fractures are associated with subsequent fractures in older age, future research could examine whether treating prediabetes before the MT reduces the risk of different fracture outcomes (eg, hip, vertebral, nonvertebral, major osteoporotic) in later life.

## Notes

Supplement.
Data Sharing Statement
