# Supplementary material for: Prediabetes and Fracture Risk Among Midlife Women in the Study of Women’s Health Across the Nation
Source: JAMA Netw Open. 2023 May 23;6(5):e2314835. doi: 10.1001/jamanetworkopen.2023.14835 (PMC10208145; doi:10.1001/jamanetworkopen.2023.14835)
Supplement: Supplement. — Data Sharing Statement [file jamanetwopen-e2314835-s001.pdf]

Data Sharing Statement

Shieh. Prediabetes and Fracture Risk Among Midlife Women in the Study of Women's Health Across the Nation. *JAMA Netw Open*. Published May 23, 2023.
doi:10.1001/jamanetworkopen.2023.14835

Data
Data available: No

Additional Information
Explanation for why data not available: Some or all datasets generated during and/or analyzed during the current study are not publicly available but are available from the corresponding author on reasonable request.
